# Magnetic Force Microscopy of Nanostructured Co/Pt Multilayer Films with Perpendicular Magnetization

**DOI:** 10.3390/ma10091034

**Published:** 2017-09-05

**Authors:** O. L. Ermolaeva, N. S. Gusev, E. V. Skorohodov, Yu. V. Petrov, M. V. Sapozhnikov, V. L. Mironov

**Affiliations:** 1 Institute for Physics of Microstructures RAS, GSP-105, 603950 Nizhny Novgorod, Russia; gusevns@ipmras.ru (N.S.G.); evgeny@ipmras.ru (E.V.S.); msap@ipimras.ru (M.V.S.); mironov@ipmras.ru (V.L.M.); 2 Saint Petersburg State University, University Embankment, 7/9, 199034 St. Petersburg, Russia; y.petrov@spbu.ru

**Keywords:** perpendicular anisotropy, magnetic domains, magnetic nanostructures, magnetic nanowires, magnetic force microscopy

## Abstract

We present the results of magnetic force microscopy investigations of domain structures in multilayer [Co (0.5 nm)/Pt (1 nm)]_5_ thin film structures (denoted hereafter as Co/Pt) modified by additional Co capping layers and by ion irradiation. It is demonstrated that a Co capping layer essentially changes the domain structure and decreases the threshold of magnetization reversal, due to the formation of noncollinear magnetization in Co/Pt. It is shown that local irradiation with a focused He^+^ ion beam enables the formation of regions with decreased easy-axis anisotropy (magnetic bubbles) that have the inverse magnetization direction in the demagnetized state of Co/Pt. The experimental results demonstrate that the magnetic bubbles can be switched using a probe of a magnetic force microscope. The possible application of these effects for the development of magnetic logic and data storage systems is discussed.

## 1. Introduction

The engineering of magnetic states in ferromagnetic nanosystems is one of the topical problems closely connected with the development of modern magnetic data processing systems and element base of spin electronics [1]. One effective solution to this problem is the creation of planar patterned ferromagnetic nanostructures using lithographic methods. The control of shape and size of the patterned elements allows ferromagnetic nanostructures with various distributions of magnetization to be obtained [2,3,4,5,6,7]. Additional possibilities are opened with the use of magnetic films with perpendicular (easy-axis) anisotropy. The fabrication of nanostructures based on multilayer systems consisting of films with easy-axis and easy-plane anisotropy enables the realization of structures with essentially noncollinear magnetization distribution [8,9,10] for magnetic devices with vertical architectures. Additionally, in recent years, new approaches to modifying the properties of magnetic films by focused ion beams have been developed [11,12,13,14,15]. These methods enable the local modification of the magnetic properties of thin-film structures without changing the surface relief. The combination of all these methods is a powerful tool for micromagnetic engineering.

In the present study, we investigate the possibilities of local modification of the domain structure in Co/Pt multilayer films with perpendicular anisotropy through the growth of additional patterned Co layers, and by local modification of the Co/Pt anisotropy parameter using a focused He^+^ ion beam.

## 2. Experiment

Multilayer thin film structures [Co (0.5 nm)/Pt (1 nm)]_5_ were grown by DC magnetron sputtering on Si(100) substrate with Ta(10 nm)/Pt(10 nm) buffer sublayer. Additionally, we deposited Co capping layers with variable thickness. The Co capping layers were patterned using lithographic methods to locally change the domain structure. The Carl Zeiss “Supra 50VP” scanning electron microscope with “ELPHY Plus” exposure system (Carl Zeiss company, Jena, Germany) was used for the e-beam lithography [4,16]. Additionally, we studied the Co/Pt structures when spatially modified by a focused ion beam. The Carl Zeiss “Orion” He^+^ ion microscope, equipped with “Nanomaker” pattern generator (Carl Zeiss company, Jena, Germany), was used to fabricate arrays of modified areas in Co/Pt [17]. 

The magnetic properties of the samples were investigated by home-made magneto-optical Kerr effect (MOKE) technique. Domain structure was studied by magnetic force microscope (MFM) “Solver HV” (NT-MDT company, Moscow, Zelenograd, Russia) equipped by DC magnet with 500 Oe perpendicular (relatively to the sample) magnetic field. The MFM probes were standard NSG-11 cantilevers coated with Co with a thickness of 30 nm. Before the measurements, the probes were magnetized along the tip axes in a 1T external magnetic field. The MFM measurements were performed both in two-pass tapping mode and in noncontact constant-height mode. The phase shift of cantilever oscillations under the gradient of the sample magnetic force was registered as the MFM contrast. The MFM measurements were performed in a vacuum chamber with the rest gas pressure of 10−4 Torr that improved the MFM signal due to an increase of cantilever quality factor.

## 3. Results and Discussion

**Figure 1:**
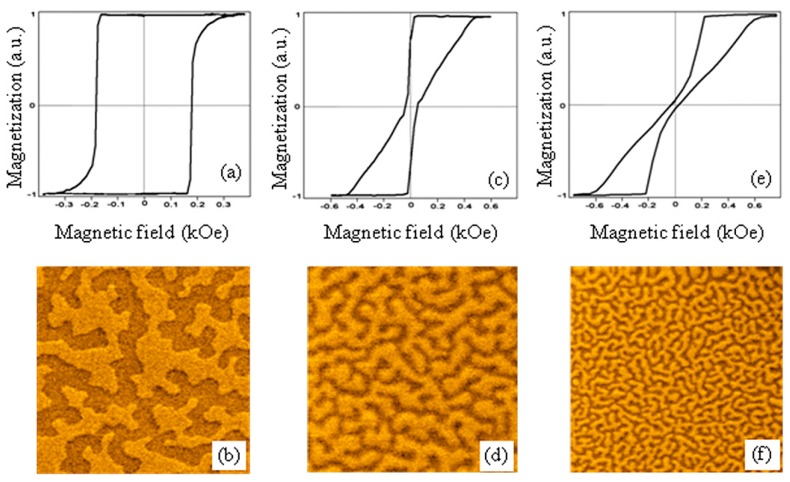
The normed MOKE hysteresis curves and MFM images of the samples’ domain structure in demagnetized state. (**a**,**b**) are for Co/Pt structure without capping layer; (**c**,**d**) are for the Co/Pt structure with 1 nm Co capping layer; (**e**,**f**) are for the Co/Pt structure with 1.3 nm Co capping layer. The MFM frame size is 5 μm × 5 μm. The MFM contrast is normalized to the maximum.

The typical hysteresis loop (the dependence of perpendicular component of magnetization on an out-of-plane external magnetic field) of the initial Co/Pt film is presented in Figure 1a. This curve has a practically rectangular shape with the remanent magnetization equal to the magnetization in saturation *M*_0_. The coercive field is about 200 Oe. The MFM image of Co/Pt labyrinth domain structure in a demagnetized state (demagnetizing field was 200 Oe) is shown in Figure 1b. 

As can be seen from Figure 1b, the typical lateral size of magnetic domains is about 1 μm. The additional Co capping layer changes the domain structure considerably. The hysteresis curve and MFM image for Co/Pt structure with 1 nm Co capping layer are represented in Figure 1c,d. In this case, we observe the narrowing of the hysteresis loop (the remanent magnetization is 0.8 *M*_0_ and the coercive field is 50 Oe) and a reduction in the size of the domains (typical width is about 350 nm). In the case of the 1.3 nm Co capping layer, the remanent magnetization is 0.2 *M*_0_, the coercive field is 10 Oe and the typical domain width is 150 nm. The decrease of the domain structure scales with increasing thickness of the Co capping layer can be explained by effects of exchange interaction. The Co-Co/Pt exchange coupling leads to a decrease in the effective perpendicular anisotropy, and to an increase in the density of the domain walls in the Co/Pt subsystem. Similar effects of changes in the domain structure were mentioned in [18], where Co/Pd structures with a NiFe capping layer were studied.

**Figure 2:**
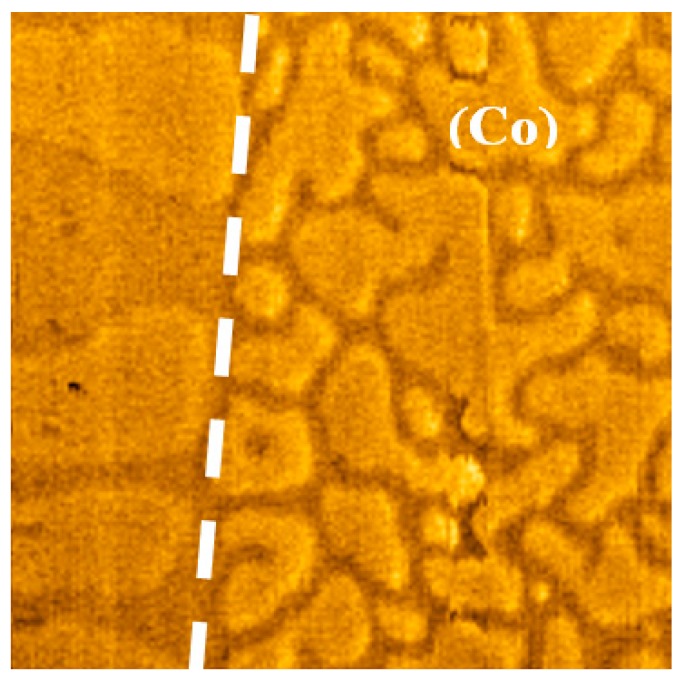
The MFM image of domain structure in demagnetized state of Co/Pt-Co (1 nm) sample near the edge of Co (1 nm) capping layer (Co capping layer is on the right side). The border between Co/Pt and Co/Pt-Co structures is shown by a dashed line. The MFM frame size is 4 μm × 4 μm. The MFM contrast is normalized to the maximum.

To create laterally inhomogeneous domain structures in the Co/Pt sample, we fabricated spatially inhomogeneous Co coatings. As an example, Figure 2 shows the MFM image of the Co/Pt-Co structure in the region near the edge of the 1 nm Co capping layer.

It can clearly be seen that the deposition of a thin Co film substantially changes the lateral domain structure of the Co/Pt. From this point of view, the patterning of the upper Co layer enables the control of the domain structure of Co/Pt layers on small spatial scales.

**Figure 3:**
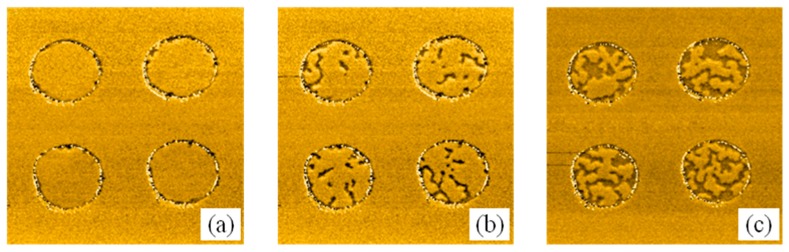
The sequential MFM images of first stage of magnetization reversal for Co/Pt sample with Co (1.5 nm) discs. (**a**) Initial state; (**b**) The remanent state after applying a 100 Oe reversed magnetic field; (**c**) The state after applying a 150 Oe magnetic field. The MFM frame size is 7 μm × 7 μm. The MFM contrast is normalized to the maximum.

**Figure 4:**
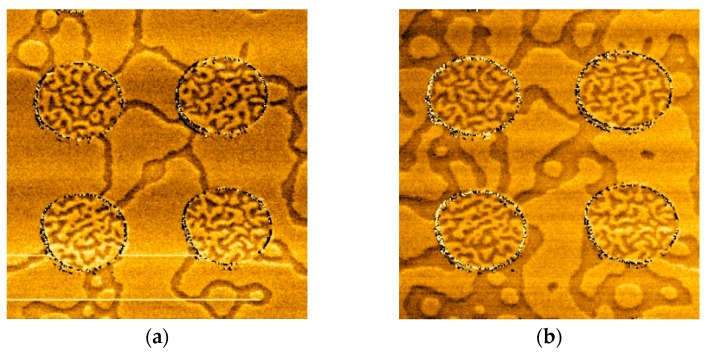
The MFM images of the second stage of magnetization reversal for Co/Pt sample with Co (1.5 nm) discs. (**a**) The remanent state after applying a 200 Oe reversed magnetic field; (**b**) The state after applying a 250 Oe magnetic field. The MFM frame size is 7 μm × 7 μm. The MFM contrast is normalized to the maximum.

We investigated the change of magnetic domains caused by circular Co islands covering the Co/Pt. In Figure 3 and Figure 4 we show the sequence of MFM images during magnetization reversal of the Co/Pt film with Co circular discs (1.5 nm of thickness and 2 μm in diameter) in an external perpendicular magnetic field. 

Initially, the sample was uniformly magnetized in an external field of 500 Oe. The MFM image of the initial state is shown in Figure 3a. The contours of the discs are visible due to the influence of the edge relief on the MFM image. The magnetization reversal of this sample occurred in two stages. In the first stage (in the fields less than the CoPt coercive field of 200 Oe), we observed the remagnetization of the regions of Co/Pt under the Co disks (Figure 3b,c). At the second stage, in the fields exceeding 200 Oe, the magnetization was reversed outside the discs (Figure 4a,b).

**Figure 5:**
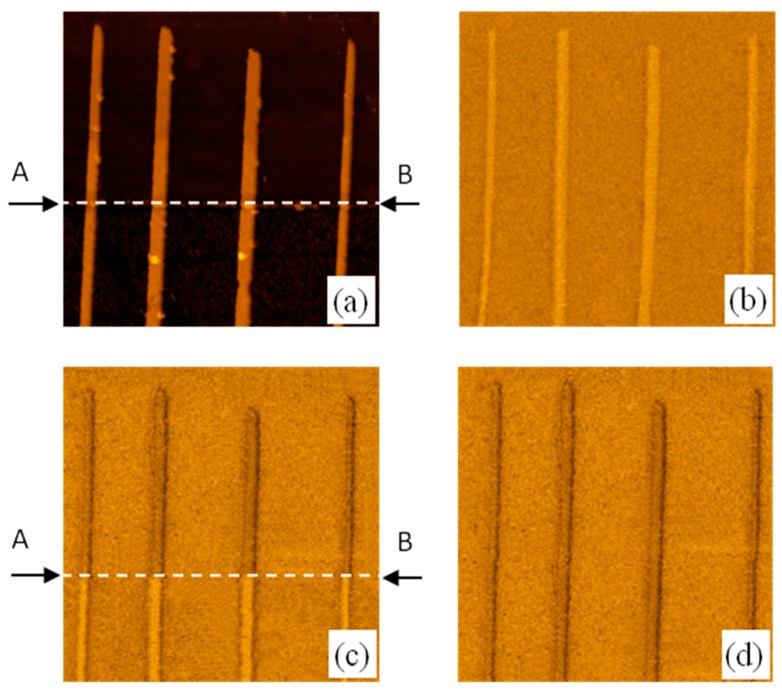
The sequential stages of remagnetization for Co/Pt NWs partly covered by Co capping layer. (**a**) The AFM image of the sample with Co/Pt NWs. The area below line A-B is covered by Co (1.3 nm) capping layer. The border of Co capping layer is shown by arrows and dashed line; (**b**) The MFM image of up-magnetized sample; (**c**) The MFM image of partly remagnetized NWs after applying of 150 Oe reversed magnetic field; (**d**) The MFM image of down-magnetized NWs in the field of 200 Oe. The frame sizes are 1.5 μm × 1.5 μm. The MFM contrast is normalized to the maximum.

We applied these effects in order to control the domain structure in planar Co/Pt nanowires (NWs). The NWs were fabricated by e-beam lithography and Ar^+^ ion etching [16]. The lateral sizes of the NWs were 100 nm × 3000 nm. The atomic force microscopy (AFM) image of the NW array is presented in Figure 5a. 

The NW array was partly covered by a Co (1.3 nm) layer (the border of Co layer is indicated by arrows and a dashed line in Figure 5a). We investigated the magnetization reversal of this sample in an external perpendicular magnetic field. The sequential stages of remagnetization are presented in Figure 5b–d. In the initial state, all NWs were magnetized uniformly (Figure 5b) in the upward directed 500 Oe magnetic field. Afterward we applied a 150 Oe reversed field (*H*_1_), and observed a partial remagnetization of the NWs in remanent state (Figure 5c). This state is stabilized by magnetostatic interaction between parts of the NWs having opposite magnetization. When we applied a 200 Oe magnetic field (*H*_2_) all NWs were remagnetized completely. The magnitude of the gap Δ*H* = *H*_2_ − *H*_1_ was equal to 50 Oe, which is quite enough for applications in a variety of devices based on NW remagnetization. 

Another method for local modification of the magnetic properties of Co/Pt films is their irradiation by focused ion beams [17,19,20]. Depending on the dose of the irradiation, the perpendicular easy-axis magnetic anisotropy can be reduced or even transformed into easy-plane anisotropy. This method allows the formation of submicron structures with different topologies [19,20].

**Figure 6:**
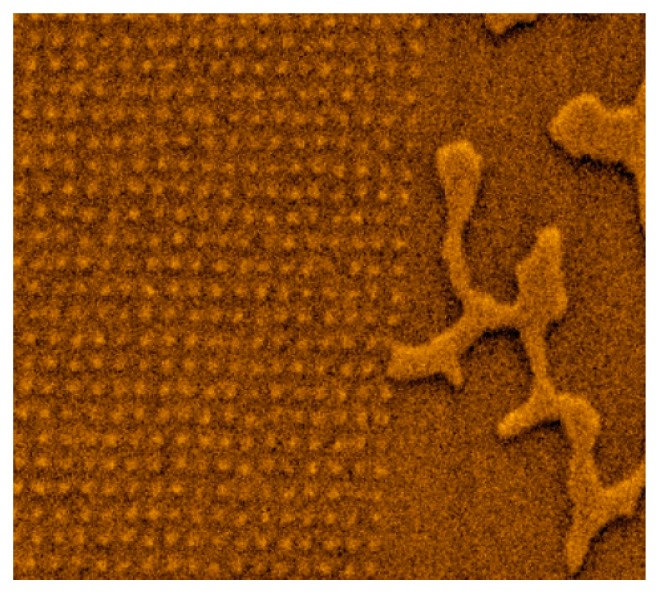
The MFM image of the Co/Pt film locally irradiated by focused He^+^ ion beam. The irradiated area is on the left side, unirradiated area is on the right side of the picture. The MFM frame size is 5 μm × 5 μm. The MFM contrast is normalized to the maximum.

We investigated Co/Pt film with an array of locally irradiated spots of 100 nm in diameter, ordered in a square lattice with a 200 nm period. The domain structure of the Co/Pt sample (in demagnetized state after applying a 200 Oe reversed magnetic field) near the boundary between the irradiated and unirradiated regions is shown in Figure 6.

It can clearly be seen that periodic ion irradiation essentially changes the spatial structure of magnetization, and allows the realization of high-density lattices of inverted domains (bubbles). These objects can be used for application in data storage systems. 

**Figure 7:**
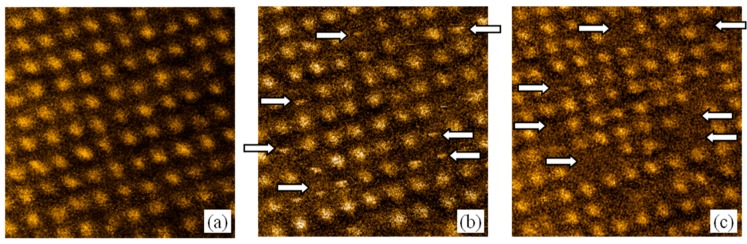
The successive MFM images of magnetic bubble array in Co/Pt. (**a**) The initial state of uniformly magnetized array; (**b**) The MFM imaging with low scanning height. The places of sharp switching of MFM contrast are indicated by white arrows; (**c**) The final state of magnetic bubble array after the MFM probe switching. The switched bubbles are indicated by white arrows. The MFM frame size is 2 μm × 2 μm. The MFM contrast is normalized to the maximum.

The effects of magnetization reversal in the arrays of Co/Pt magnetic bubbles under an external magnetic field were investigated earlier in [17]. Here, we studied the switching of bubbles under the influence of an inhomogeneous stray field of the MFM probe. The successive MFM images of the same region of bubble array are presented in Figure 7. Initially, the sample was magnetized downward in the 500 Oe external field. Afterward we applied 200 Oe reversed magnetic field to lead the sample in demagnetized state. All bubbles had upward-directed magnetic moments (Figure 7a), while the MFM probe had magnetic moments directed in the opposite direction. Nonperturbing MFM imaging was carried out in constant-height mode with a scanning height of about 30 nm (the amplitude of oscillation was 10 nm). At the second stage, we reduced the scanning height to 15 nm to observe the switching of bubble magnetization under the MFM probe field. The corresponding MFM image is shown in Figure 7b. Some cases of sharp changing of MFM contrast during the scanning are indicated by arrows. As a result, we observed the switching of contrast (disappearance of white dots) in some areas of the MFM image (Figure 7c). The mechanism of bubble switching is similar to the mechanism for changing the magnetization of Co/Pt discs described in Ref. [21]. Through manipulation of the probe position over the sample [22], it is possible to realize the selective magnetization reversal of individual bubbles.

## 4. Conclusions

We have studied the peculiarities of domain structure in multilayer Co/Pt thin-films modified by patterned Co capping layers and by a focused He^+^ ion beam. It was shown that the application of an additional Co layer allows a reduction in the characteristic scale of the domain structure and the magnitude of magnetization reversal fields. Therefore, the nanostructuring of Co/Pt films and covering Co layers enables the possibility to control domain wall nucleation and pinning/depinning processes for the development of magnetic devices. On the other hand, the application of local irradiation of Co/Pt films by ion beams opens up significant opportunities for creating highly dense arrays of controllable magnetic domains that can be used to develop novel and promising data storage systems.
